# Fine-mapping of genetic loci driving spontaneous clearance of hepatitis C virus infection

**DOI:** 10.1038/s41598-017-16011-2

**Published:** 2017-11-20

**Authors:** Hailiang Huang, Priya Duggal, Chloe L. Thio, Rachel Latanich, James J. Goedert, Alessandra Mangia, Andrea L. Cox, Gregory D. Kirk, Shruti Mehta, Jasneet Aneja, Laurent Alric, Sharyne M. Donfield, Matthew E. Cramp, Salim I. Khakoo, Leslie H. Tobler, Michael Busch, Graeme J. Alexander, Hugo R. Rosen, Brian R. Edlin, Florencia P. Segal, Georg M. Lauer, David L. Thomas, Mark J. Daly, Raymond T. Chung, Arthur Y. Kim

**Affiliations:** 1 Analytic and Translational Genetics Unit, Massachusetts General Hospital, Boston, MA 02114 USA; 2 Stanley Center for Psychiatric Research, Broad Institute of MIT and Harvard, Cambridge, MA 02142 USA; 3 Department of Medicine, Massachusetts General Hospital, Harvard Medical School, Boston, MA 02114 USA; 4 Johns Hopkins University Bloomberg School of Public Health, Baltimore, MD 21205 USA; 5 Johns Hopkins University School of Medicine, Baltimore, MD 21205 USA; 6 Division of Cancer Epidemiology and Genetics, National Cancer Institute, Rockville, MD 20852 USA; 7 IRCCS Casa Sollievo della Sofferenza Hospital, San Giovanni Rotondo, Italy; 8 Department of Medicine, Purpan Hospital, University of Toulouse III, Toulouse, France; 9 Rho, Chapel Hill, NC 27517 USA; 10 South West Liver Unit, Plymouth Hospitals NHS Trust, Plymouth, United Kingdom; 11 Henry Welcome Laboratories, Southampton General Hospital, Southampton, UK; 12 University of California and Blood Systems Research Institute, San Francisco, CA 94118 USA; 13 Cambridge University Hospitals NHS Foundation Trust and Addenbrooke’s Hospital, Cambridge, United Kingdom; 14 University of Colorado, Aurora, Colorado, 90045 United States; 15 State University of New York Downstate College of Medicine, Brooklyn, New York, USA; 16 Brigham and Women’s Hospital, 75 Francis Street, Boston, MA 02115 USA

## Abstract

Approximately three quarters of acute hepatitis C (HCV) infections evolve to a chronic state, while one quarter are spontaneously cleared. Genetic predispositions strongly contribute to the development of chronicity. We have conducted a genome-wide association study to identify genomic variants underlying HCV spontaneous clearance using ImmunoChip in European and African ancestries. We confirmed two previously reported significant associations, in the *IL28B/IFNL4* and the major histocompatibility complex (MHC) regions, with spontaneous clearance in the European population. We further fine-mapped the association in the MHC to a region of about 50 kilo base pairs, down from 1 mega base pairs in the previous study. Additional analyses suggested that the association in MHC is stronger in samples from North America than those from Europe.

## Introduction

The development of chronic viral infection represents a failure to mount an adequate innate and/or adaptive response to a specific pathogen. Infection with hepatitis C virus (HCV) in humans represents a paradigm of a dichotomous outcome of infection, as approximately three quarters of acute HCV infections evolve to a chronic state, but one quarter are spontaneously cleared^1^. As such, it is likely that genetic predispositions, especially at loci that regulate the innate and/or adaptive immune response, strongly contribute to the development of chronicity. A prior genome wide association study (GWAS) conducted by our consortium demonstrated striking associations of spontaneous resolution of HCV with polymorphisms near the IFN-L3 locus (*IL28B*) and in the HLA class II locus (Duggal *et al*.^2^).

The associations identified in Duggal *et al*. span large genomic regions, specifically 55,000 base pairs for the Il28B locus and >1 mega base pairs for the HLA class II locus. Recently advances in genomic technologies allowed a more precise characterization of genetic associations and facilitated resolving these associations to much smaller genomic regions. Firstly, ImmunoChip^3^, a customized array platform with deeper coverage of loci enriched in autoimmune diseases, provides coverage of additional genomic variants for an opportunity to explore with greater precision the contribution of these loci to the clearance of viral infection. Secondly, additional coverage of the MHC region can be gained using an imputation algorithm that takes into account the long range linkage-disequilibrium in MHC, and a large customized reference panel with improved coverage of the MHC region^4^. Thirdly, fine-mapping algorithms^5,6^, designed with the goal to resolve known genetic associations to smaller sets of variants, can be used with the high density genomic data to further improve the precision of the genetic associations.

We therefore conducted an analysis of a large pool of spontaneous resolvers and chronic patients of HCV using the ImmunoChip platform, the SNP2HLA algorithm with the T1DGC MHC imputation reference panel^4^, and a recently-developed fine-mapping algorithm^6,7^ to (1) more precisely define the susceptible variant within the known associated loci; and (2) identify additional loci associated with clearance. Similar successes have been achieved in other conditions such as inflammatory bowel diseases^6,8^. Additionally, we explored the hypothesis that there are shared mechanisms that define a “brisk” immunity able to confer both susceptibility to autoimmune disease and improved control of pathogens. We also examined the influence of region (North America versus European) upon associations with HLA within the European ancestry, as previous studies have shown variability of results, especially for the class I locus^9–12^.

## Results

**Figure 1:**
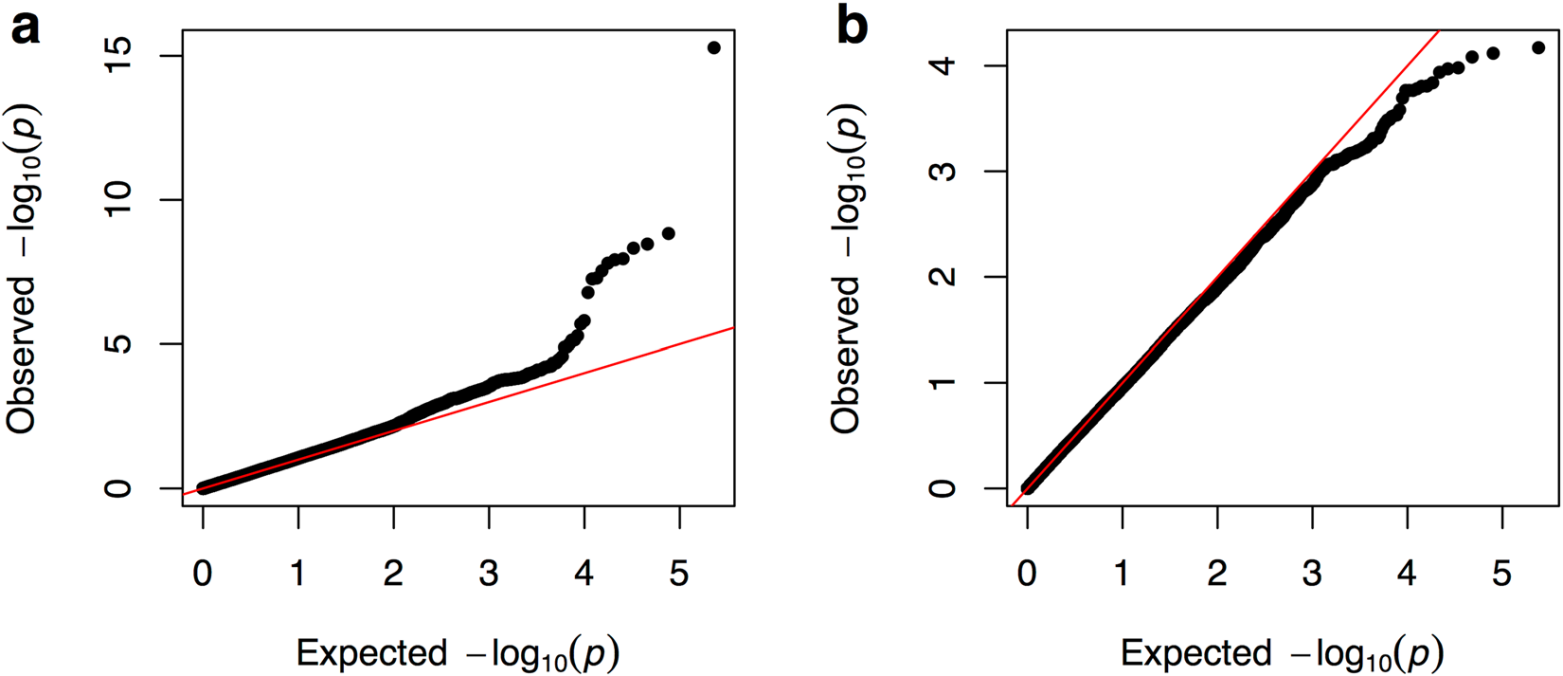
QQ plot for cohorts of European (**a**) and African (**b**) ancestries. The red line indicates the null distribution. Only variants with minor allele frequency >2% were used in this figure.

The final dataset after QC has 166,537 variants for 527 cases/828 controls of European ancestry; and 171,161 variants for 75 cases/171 controls of African ancestry (Table 1). For each ancestry, we performed logistic regression under the additive model using the first two principal components as covariates. The QQ plot (Fig. 1, using common variants with >2% minor allele frequency) and the genomic control (GC) factors (0.98 for the European ancestry and 0.92 for the African ancestry using designated null variants) indicate the effective control of the population stratification.

**Table 1:** Variants and samples in this study.

	European ancestry	African ancestry
Variants		
Initial	191,357	191,357
HWE (p-value < 1E–6)	−394	−300
Missingness (5%)	−24,426	−19,896
After QC	166,537	171,161
Samples		
Initial	1,416	227
Missingness (5%)	−24	−2
Heterozygosity	−9	−1
Duplicated	−28	−6
After QC	527 cases/828 controls	75 cases/171 controls

Negative numbers indicate numbers of variants or samples removed.

**Figure 2:**
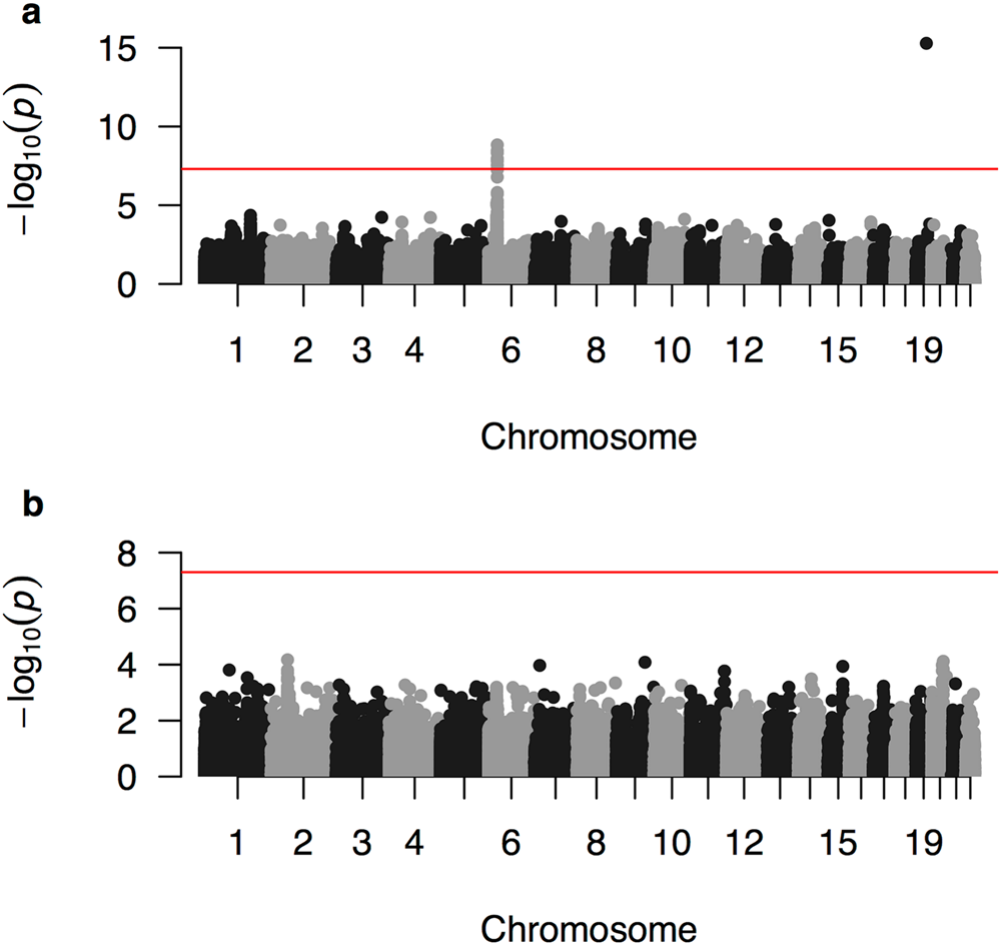
Manhattan plot for cohorts of European (**a**) and African (**b**) ancestries. The red horizontal line indicates the genome-wide significance threshold.

For European samples, we identified 8 genome-wide significant variants (p-value < 5E-8) in two loci (Fig. 2 and Table 2). The variant on chromosome 19, rs8099917, shows the strongest association with spontaneous clearance (p-value = 5E-16). Patients carrying the minor allele, G, are on average 2.5x (odds ratio = 0.39) less likely to spontaneously clear the virus compared to those with two copies of the C allele. This variant is roughly 7,000 base pairs upstream of the *IL28B* gene, and has been previously reported to be associated with HCV spontaneous clearance^2^ and the response to chronic HCV therapy in Asian populations^13^. Previous studies have also shown an association between *IL28B* and interferon-based clearance of HCV^14^, and an association between a frameshift variant upstream of *IL28B* and impaired clearance of hepatitis C virus^15^. Because the *IL28B/IFLN4* region was not designed as a high-density locus in ImmunoChip, we could not test other variants in this region for their association with HCV spontaneous clearance, and was unable to provide a better resolution in this locus.

**Table 2:** Genome-wide significant associations.

CHR	SNP	POSITION	Tested Allele	European ancestry		African ancestry	
				OR	p-value	OR	p-value
19	rs8099917	39743165	G	0.385	5.24E-16	0.555	0.2309
6	rs6457620	32663999	G	0.605	1.47E-09	0.758	0.1571
6	rs6457617	32663851	C	0.614	3.43E-09	0.758	0.1573
6	rs9275224	32659878	A	0.615	4.76E-09	0.685	0.0550
6	rs6932517	32678182	C	0.600	1.10E-08	0.557	0.0064
6	rs9357152	32664960	G	1.664	1.20E-08	1.484	0.1075
6	rs9378125	32679732	G	1.657	1.57E-08	1.437	0.1397
6	rs2858324	32660375	A	0.604	2.89E-08	0.590	0.0178

List of variants that have genome-wide significant association with HCV spontaneous clearance (before imputation). The genomic position is in HG18.

**Figure 3:**
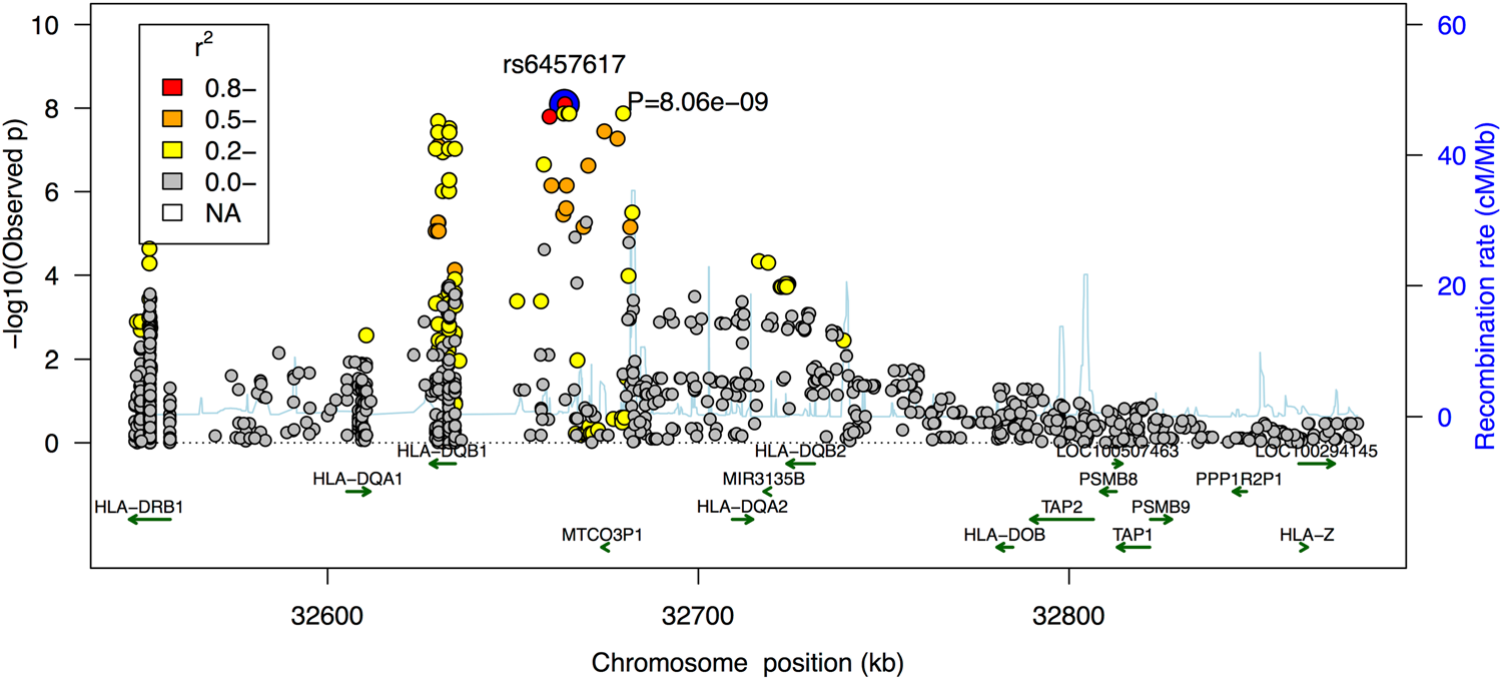
Regional association plot for the MHC class II region. Color indicates the linkage equilibrium with the top associated variant (rs6457620).

The other genome-wide significant locus for the European samples is the major histocompatibility complex (MHC) locus. Genome-wide significant variants in this region are reported in Table 2 (before imputation). We used SNP2HLA^4^ and a customized reference panel from a T1D study to impute missing variants, HLA alleles and amino acid residues for this region. We identified 12 SNPs and 5 amino acids that are genome-wide significant (Fig. 3 and Table 3, boldfaced). No secondary signal in this region exceeded the suggestive significance threshold (1 × 10^−5^) after conditioning on the primary signal. Therefore, all variants reported in Table 3 account for the same association signal. Using a fine-mapping algorithm described in another study^6,7^, we constructed the 99% credible set, which is a set of variants that has 99% probability of having the causal variant in this locus (Table 3, full). Comparing with the previous study^2^ which identified this association to a region of more than 1 mega base pairs, we mapped this association to a much smaller region of 50,562 base pairs.

**Table 3:** Associations in the 99% credible set in the MHC region.

CHR	SNP	POSITION	Tested Allele	OR	P	Probability
6	**rs6457617**	32771829	C	0.6172	8.06E-09	0.1197
6	**rs6457620**	32771977	G	0.6172	8.06E-09	0.1197
6	**rs9378125**	32787710	G	1.664	1.34E-08	0.0732
6	**rs5000632**	32771666	G	1.665	1.35E-08	0.0727
6	**rs9357152**	32772938	G	1.665	1.35E-08	0.0727
6	**rs9394113**	32773145	G	1.665	1.35E-08	0.0727
6	**rs9275224**	32767856	A	0.6229	1.60E-08	0.0616
6	**AA_DQB1_167_32737787_R**	32737787	A	1.717	2.05E-08	0.0483
6	**AA_DQB1_13_32740798_G**	32740798	A	1.708	3.03E-08	0.0331
6	**rs9275516**	32782621	A	0.6079	3.60E-08	0.0280
6	**SNP_DQB1_32737787**	32737787	T	1.7	3.79E-08	0.0266
6	**SNP_DQB1_32740798**	32740798	G	1.7	3.79E-08	0.0266
6	**SNP_DQB1_32740759_T**	32740759	P	1.7	3.79E-08	0.0266
6	**SNP_DQB1_32740760_A**	32740760	P	1.7	3.79E-08	0.0266
6	**AA_DQB1_13_32740798_A**	32740798	P	1.7	3.79E-08	0.0266
6	**AA_DQB1_26_32740759_Y**	32740759	P	1.7	3.79E-08	0.0266
6	**AA_DQB1_167_32737787_H**	32737787	P	1.7	3.79E-08	0.0266
6	rs6932517	32786160	C	0.6123	5.38E-08	0.0190
6	SNP_DQB1_32737837	32737837	A	0.627	8.09E-08	0.0128
6	SNP_DQB1_32737148	32737148	G	1.68	9.42E-08	0.0110
6	AA_DQB1_45_32740702	32740702	E	1.68	9.42E-08	0.0110
6	SNP_DQB1_32740702	32740702	T	1.68	9.42E-08	0.0110
6	SNP_DQB1_32742309_A	32742309	P	1.68	9.42E-08	0.0110
6	HLA_DQB1_0301	32739039	P	1.675	1.14E-07	0.0092
6	rs9469220	32766288	G	0.6393	2.24E-07	0.0048
6	rs2856717	32778286	T	0.6255	2.38E-07	0.0045
6	AA_DQB1_26_32740759_L	32740759	A	1.534	5.32E-07	0.0021
6	SNP_DQB1_32740759_A	32740759	A	1.534	5.32E-07	0.0021
6	SNP_DQB1_32740760_G	32740760	A	1.534	5.32E-07	0.0021
6	rs2858324	32768353	T	0.6397	7.11E-07	0.0016
6	rs2647012	32772436	A	0.6397	7.11E-07	0.0016

List of variants and HLA alleles in the MHC locus that are in the 99% credible set. Genome-wide significant variants, HLA alleles and amino acid residues are boldfaced.

Neither the MHC nor the *IL28B* locus was genome-wide significant in the African ancestry. Using the heterogeneity test (fixed-effect, implemented in the R metafor package), we found that neither the MHC locus nor the *IL28B* locus have significantly different effect size (p-values = 0.47 and 0.29 respectively) across the two populations. Therefore, the difference in the significance is likely driven by the sample size and/or the allele frequency differences.

In addition to the genome-wide significant loci, we examined genes outside the HLA that have been previously associated with HCV spontaneous clearance^16^. Only genes *IFNG-AS1* (p-value = 6E-4) and *STAT1* (p-value = 3E-4) showed marginal evidence of association (p-value < 1E-3), and this effect was observed only in the European cohort. No genes reached the marginal p-value threshold in the samples of African ancestry. *IFNG-AS1* is a long noncoding RNA that is expressed in CD4 T cells and promotes Th1 responses^17^. *STAT1* is one of the key mediators of the type I, II and III interferon responses.

**Figure 4:**
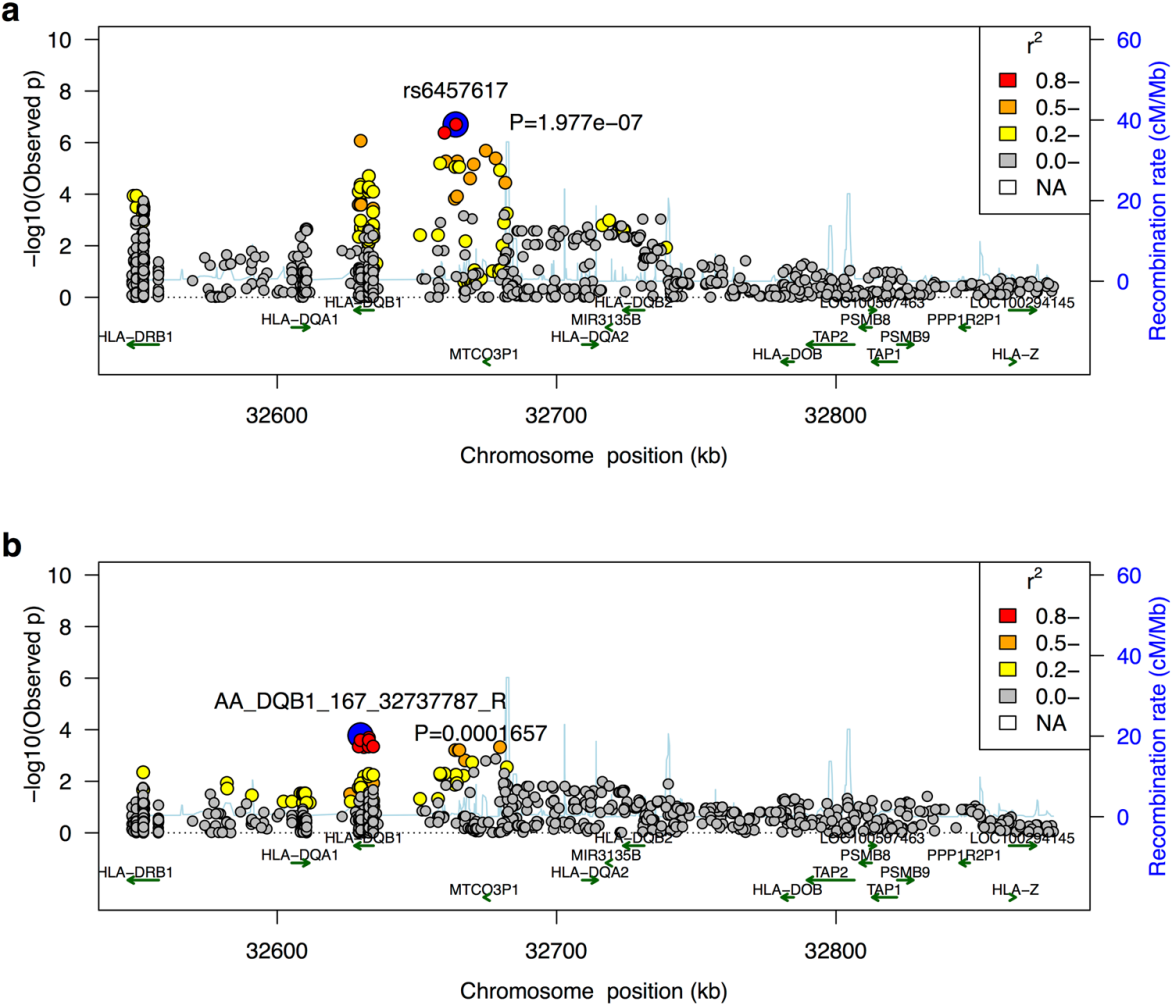
Regional association plot for the MHC class II region in North American samples (**a**) and European samples (**b**). Color indicates the linkage equilibrium with their respective top associated variant.

**Figure 5:**
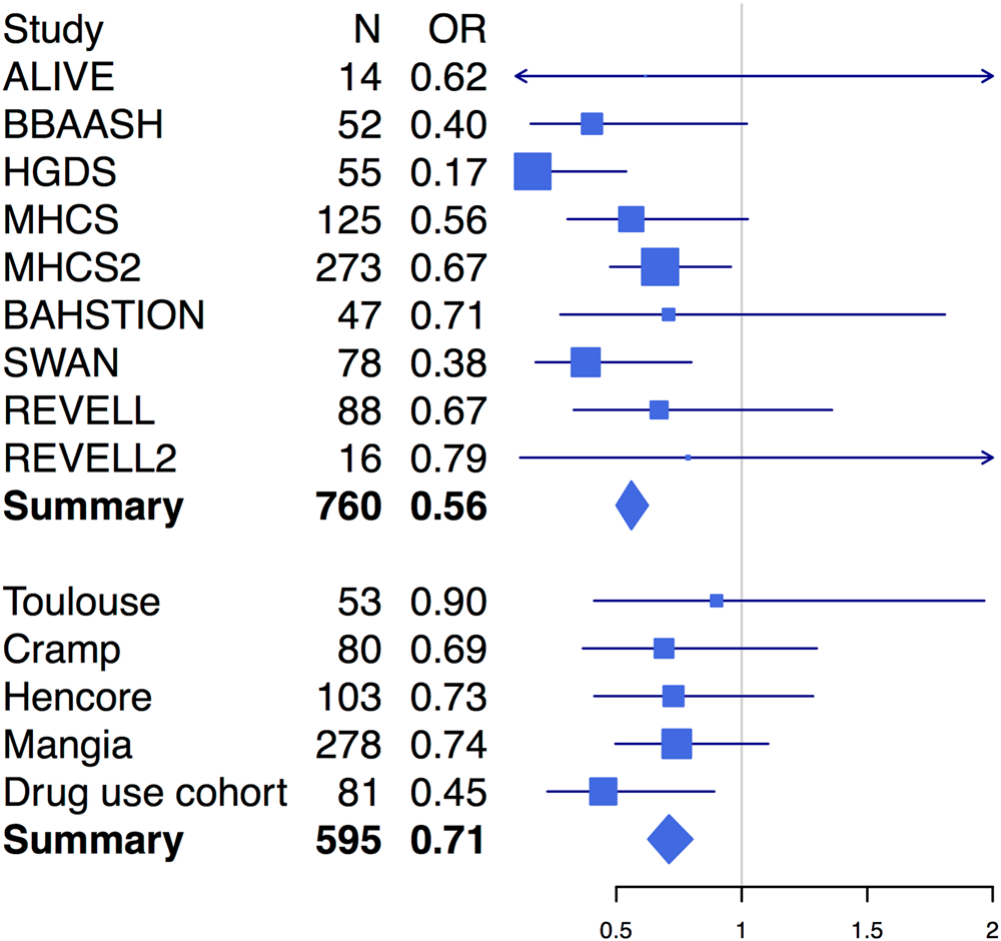
Forest plot for the top MHC class II association (rs6457620). Cohorts have been grouped by the geographical locations of where they were collected: the top panel includes cohorts collected in North America, and the bottom panel includes cohorts collected in Europe.

Since HCV is particularly diverse, with up to a 30% difference at the amino acid level between major viral genotypes, the strain of infecting virus may influence HLA-mediated clearance^11,18^. Unfortunately, information regarding the virus genotype or subtype was not available in this study so a direct comparison is therefore not possible. However, an indirect comparison is possible by taking advantage of the observation that North American patients are much more likely to be infected with the 1a virus and European patients are much more likely to be infected by the 1b virus^19^. We observed that the association in the class II MHC locus, after accounting for the sample size, age, sex and exposure (Methods), is stronger in North American samples than in European samples (Fig. 4) with marginal significance (p = 0.044). This suggests that viral subtype may have influenced the genetic mechanism underlying the clearance of HCV. Meta-analysis by cohorts confirms this observation (Fig. 5). We also interrogated the potentially protective effect of certain SNPs associated with HLA class I alleles previously implicated in spontaneous clearance. No SNP associated with class I was associated with genome-wide significance, including those associated with HLA B*27 subtypes (p-values > 0.05). The strength of association with the SNP most closely linked with HLA-B*57 and control of HIV-1 (rs2395029) was not genome-wide significant but showed a marked difference by continent (North America p-value = 8.6E-4, Europe p-value = 0.078, overall p-value = 1.0E-4), suggesting that any protective effect of this class I allele differs by region.

Autoimmune disorders have been reported to have shared genetic susceptibility loci^20,21^. For each of 5 major autoimmune diseases, including inflammatory bowel disease, systemic lupus erythematosus, rheumatoid arthritis, celiac disease and multiple sclerosis, we listed all variants that reached p-value < 0.001 (or the best variant) in this analysis. We found no shared variant after considering multiple testing. A full exploration of the hypothesis that susceptibility to autoimmunity also confers ability to clear HCV will require a larger sample size. This analysis was only performed in the European cohort because the African cohort has even less power due to the sample size, and GWAS results in samples of African ancestry for other autoimmune disorders is more limited.

An alternate approach, taken by the International Genetics of Ankylosing Spondylitis Consortium^22^, is to search for the reported associations with other diseases in loci having suggestive evidence (p-value < 1E-5), *i*.*e*., the MHC and the *IL28B* loci in this study. We only performed the search in *IL28B* because MHC has been already implicated in many autoimmune disorders. We searched within 0.5 Mb around the lead SNP (rs8099917) in *IL28B* for associations with other diseases that have been reported in the NHGRI GWAS catalog (https://www.ebi.ac.uk/gwas, accessed on July 1, 2017). This catalog hosts published associations between genetic variants and thousands of diseases/traits, including autoimmune, inflammatory, cardiovascular, metabolic, brain and diseases. Three SNPs were found to be in partial linkage disequilibrium (R^2^ > 0.4) with our lead SNP in *IL28B*, including rs12980275 (R^2^ = 0.41) associated with lipid levels in hepatitis C treatment^23^, rs12979860 (R^2^ = 0.42) associated with chronic hepatitis C infection/response to hepatitis C treatment^14^ (discussed in the previous sections), and rs688187 (R^2^ = 0.40) associated with mucinous ovarian carcinoma^24^.

## Discussion

We have conducted a genome-wide association study to identify genomic variants underlying the HCV spontaneous clearance using ImmunoChip. Consistent with previous reports^2^, two loci were found to be significantly associated with the HCV spontaneous clearance in the European cohort. The ImmunoChip design, the imputation pipeline specifically designed for the MHC region and the novel fine-mapping algorithm facilitated the accurate characterization of classical HLA types and allowed us to achieve a higher resolution in the MHC region. Twelve SNPs and 5 amino acids in the MHC region were found to be significantly associated and no secondary signal remains after conditioning on the best SNP. Fine-mapping mapped this association to a region of about 50 kilo base pairs, down from 1 mega base pairs in the previous study. This fine-mapping analysis was conducted in the European population. We note that if the MHC association is shared across populations, this fine-mapping results will also be generalizable to other populations.

We found no associated variants in the African cohort, probably due to different genetic background (in the case of the *IL28B* locus) and limited sample size (in the case of the MHC locus). Previous studies^18^ suggest that spontaneous clearance can be more common with one virus genotype than another^25^. We noted that the association in the class II MHC locus might be stronger in samples from North American than those from Europe. While viral subtyping was not available with sufficient numbers in this cohort, the virus subtype 1a is more prevalent in North America than in Europe where subtype 1b predominates. Previous studies showed that key polymorphisms between viral subtype may have influenced HLA-restricted genetic associations underlying the clearance of HCV^11,26^. In HIV-1, viral mutational escape over first decades of the epidemic reduced the protective effect of key HLA alleles on a population level^27^. For HCV, additional evidence, such as virus typing, is needed to confirm this finding.

Limitations of this study include inability to dissect SNPs near the *IL28B/IFLN4* region, as this loci had not been previously implicated in autoimmune GWAS studies. While the ImmunoChip did include rs8099917 as a surrogate for this region, additional information regarding associations with rs12979860 and ss469415590 is not available^15^. Also, this study was a fine-mapping exercise that narrowed the MHC significantly but was not fully independent due to considerable overlap with the previous GWAS.

Previous studies of GWAS data revealed that there are SNPs and loci with evidence of association across multiple immune-mediated diseases^20^. We found several variants that have suggestive and plausible evidence of associations with both HCV spontaneous clearance and another autoimmune disorder. Despite the observation that none of these variants are significant after the strict Bonferroni correction, they jointly confirm the concept that shared genetic mechanisms underlie autoimmune disorders and suggest the hypothesis that susceptibility to autoimmunity may also confer ability to clear HCV. Fuller exploration of this hypothesis will require further analyses with larger sample sizes.

## Methods

### Overview of samples

1,944 samples from 13 cohorts (ALIVE, BBAASH, HGDS, MHCS, Rosen and colleagues, REVELL, BAHSTION, SWAN, Toulouse, Cramp and colleagues, Hencore, Mangia and colleagues, UK Drug Use Cohort) were genotyped in this study, as previously described^2^. Self-clearance of HCV was coded as cases (718 samples) and persistence of HCV was coded as controls (1,180 samples). Samples with unidentified clearance status were not used (46 samples). All samples were genotyped using Illumina’s ImmunoChip, a custom Infinium chip with 196,524 SNPs and small in/dels. A large number of these variants are in 187 high-density regions known to be associated with twelve autoimmune disorders and inflammatory diseases. Variants in these high-density regions include 289 established associations, variants from 1000 genome project low coverage pilot 1 study^28^, and variants discovered in re-sequencing^29^. In addition, roughly 25,000 variants were included as replication of unrelated diseases as part of the WTCCC2 project, with the purpose of serving as null SNPs in analyses.

### Sample ethnicities

To identify the sample ethnicities, we first constructed the principal component axes using Hapmap samples. 988 founders from Hapmap phase 3 (draft release 2)^30^, including samples from ethnicities ASW, CEU, CHB, CHD, GIH, JPT, LWK, MEX, MKK, TSI and YRI were used. To calculate the principal components, only common variants that are also present in the ImmunoChip were used, and AT/GC SNPs were excluded to avoid ambiguous strand alignment. We performed LD pruning of the variants, resulting in a total of 15,525 variants used to create the principal components. The study samples were then projected to the principal component axes and assigned the ethnicities based on their distance to the Hapmap samples. Out of 1,898 samples, 1,416 samples were mapped to European ancestry, 225 samples were mapped to African ancestry and 227 samples were admixtures and were not used in this study.

### Quality control

QC was performed separately on samples of European and African ancestries separately. Variants that failed the Hardy–Weinberg equilibrium test in controls (p-value ≤ 1E-5) or had low call rate (≤95%) were identified, and 24,820 variants were removed in European samples and 20,196 variants were removed in African samples. The remaining variants were used to perform QC in samples. Samples were cleaned for having low call rate (≤95%) or having high heterozygosity rate (>3 standard deviations from the mean).

We then created a LD pruned dataset for calculating the identity by state (IBS) matrix and the principal components. We pruned the variants using a sliding window of 50 variants, step size of 5 variants and variance inflation factor threshold of 1.25. There were 20,782 variants in European samples, and 21,778 variants in African samples after the pruning. The IBS matrix was calculated using this LD pruned dataset and checked for sample relatedness. 28 duplicated samples in European cohorts and 9 duplicated samples in cohorts of African ancestry have been identified and removed (pi_hat > 0.9). The final dataset has 527 cases and 828 controls for European cohorts, and 75 cases and 171 controls for African cohorts.

To correct for within European and within African population stratification, we calculated the principal components for samples of European ancestry and African ancestry, respectively. The first two principal components sufficiently control the population stratification in both ancestries (results not shown) and were use in the association analysis as covariates.

### Imputation

Imputation of the MHC region was performed on QC cleaned data using SNP2HLA^4^. This software package takes advantage of the long-range linkage disequilibrium between HLA loci and SNP markers across the MHC region and can perform accurate imputation of classical HLA types starting from SNP genotype data. The reference panel was created using the Type 1 Diabetes Genetics Consortium’s high quality HLA reference panel (roughly 5,000 European samples), which includes classical HLA alleles and amino acids at class I (HLA-A, -B, -C) and class II (-DPA1, -DPB1, -DQA1, -DQB1, and -DRB1) loci.

### Association test

All association tests were performed in PLINK 1.07^31^ using the logistic regression. We assumed additive models and used the first two principal components as covariates in the regression. HCV spontaneous clearance was coded as case so an odds ratio >1 indicates the tested allele increases the probability of spontaneous clearance.

### Test of heterogeneity across North America and Europe samples

To evaluate whether the effect of the MHC association is consistent across samples from North America and Europe, we conducted the association test with age, gender and the HCV exposure (IDU v.s. non-IDU) as covariates to control for potential confounding. We only used samples that have non-missing measurements in these variables. For North America samples, we have 173 cases (spontaneous clearance) and 298 controls; and for Europe samples we have 144 cases and 266 controls. The heterogeneity test was conducted using the odds ratio and standard error from the association test in a fixed-effect model implemented in the R metafor package.

### Use of experimental animals, and human participants

No experimental animals were used in this study. The study protocols were approved by the institutional review board (IRB) at each center involved with recruitment (listed at the end). Informed consent and permission to share the data were obtained from all subjects, in compliance with the guidelines specified by the recruiting center’s IRB. All experiments were performed in accordance with relevant guidelines and regulations.

- Massachusetts General Hospital
- Johns Hopkins School of Medicine
- Division of Cancer Epidemiology and Genetics, National Cancer Institute
- Casa Solievo della Sofferenza Hospital, Italy
- Imperial College London
- Toulouse III University, France
- Plymouth Hospitals, UK
- Weill Cornell Medical College
- Blood Systems Research Institute
- University of Cambridge
- University of Colorado

### Data availability

The data that support the findings of this study are available from the corresponding authors but restrictions apply to the availability of these data, which were used under license for the current study, and so are not publicly available. Data are however available from the authors upon reasonable request.
